# Invariant Spatial Pattern Across Mediterranean Scrublands in the Iberian Pear (*Pyrus bourgaeana*)

**DOI:** 10.1002/ece3.70757

**Published:** 2025-01-18

**Authors:** Brayan Morera, Pedro J. Garrote, Thorsten Wiegand, Daniel Ayllón, Jose M. Fedriani

**Affiliations:** ^1^ Centro de Investigaciones sobre Desertificación CIDE CSIC‐UVEG‐GV Valencia Spain; ^2^ Estación Biológica de Doñana (EBD–CSIC) Seville Spain; ^3^ Department of Ecological Modelling Helmholtz Centre for Environmental Research‐UFZ Leipzig Germany; ^4^ German Centre for Integrative Biodiversity Research (iDiv) Halle‐Jena‐Leipzig Leipzig Germany; ^5^ Department of Biodiversity, Ecology and Evolution Complutense University of Madrid (UCM) Madrid Spain

**Keywords:** individual‐based modeling, Mediterranean landscapes, plant ecology, spatial aggregation, spatial point pattern analysis, spatially explicit modeling

## Abstract

The spatial distribution pattern of plant species is frequently driven by a combination of biotic and abiotic factors that jointly influence the arrival, establishment, and reproduction of plants. Comparing the spatial distribution of a target plant species in different populations represents a robust approach to identify the underlying mechanisms. We mapped all reproductive individuals of the Iberian pear (*Pyrus bourgaeana*) in five plots (1.39–8.57 km^2^) differing in the activity of seed dispersers and vertebrate herbivores in southern Iberian Peninsula. We used Thomas point process models to quantify the consistency in the spatial pattern and the level of spatial aggregation of this mammal‐dispersed tree among the five populations. We tested two hypotheses: (i) because the clumped defecation behavior of some dispersers can lead to local tree aggregation, and because denser groups of fruiting trees can limit seed dispersal by attracting frugivores to specific sites, we expected a consistent small‐scale aggregation pattern across all populations; and (ii) because ungulates reduce recruitment by preying on seeds and seedlings, we hypothesize that ungulate activity will show negative relationships with tree density and level of aggregation. Our spatial analysis revealed consistent and highly aggregated small‐scale patterns of all Iberian pear populations, with one critical scale aggregation, a low density of clusters and high variability in the number of trees per cluster. Ungulate activity and the number of trees per cluster showed a marginally significant negative correlation, suggesting that in areas with higher ungulate activity, trees tend to form less dense clusters. Although several of the underlying processes varied greatly among the five study sites, the Iberian pear showed a relatively consistent spatial pattern with just quantitative nuances throughout the entire region. This result has significant implications for the reproductive success of the species, management strategies, and ultimately the long‐term persistence of populations.

## Graphical abstract

Mapping of five populations of the Iberian pear (*Pyrus bourgaeana*) in the southern Iberian Peninsula revealed a consistent and highly aggregated spatial pattern. This result has significant implications for the reproductive success of the species, management strategies, and ultimately the long‐term persistence of populations.

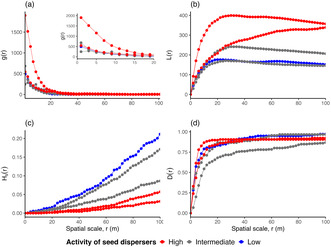

## 1 Introduction

The spatial patterns of plants within natural communities provide information about past ecological processes and are the template for future ecological and evolutionary dynamics (Law et al. 2009; Velázquez et al. 2016; Wiegand, Martínez, and Huth 2009; Wiegand et al. 2021). Furthermore, the degree to which individual plants are clustered or dispersed plays an essential role in a species' resource utilization, its role as a resource provider and its reproductive patterns (Ben‐Said 2021; Condit et al. 2000; Wiegand et al. 2007). The spatial distribution of plant species is frequently driven by a combination of biotic (e.g., seed dispersal, plant–plant facilitation, and/or competition) and abiotic factors (e.g., water, nutrient, and light availability) that jointly influence the arrival, establishment, and reproduction of plants (Brooker et al. 2008; Callaway 2007; Filazzola and Lortie 2014). Plant populations usually show patterns of spatial aggregation (Condit et al. 2000; Garrote, Castilla, and Fedriani 2019; Wiegand et al. 2007, 2021), which can be related to several non–mutually exclusive processes, including (i) seed dispersal limitation (Rodríguez‐Pérez, Wiegand, and Traveset 2012; Shen et al. 2013; Wiegand et al. 2007, 2021), (ii) clumped animal seed dispersal (Beckman, Neuhauser, and Muller‐Landau 2012; Howe 1989), (iii) spatial heterogeneity of environmental conditions and resources (e.g., Getzin et al. 2008; Shen et al. 2013), (iv) asexual reproduction (Castilla et al. 2019), and (v) plant–plant facilitation (Garrote, Castilla, and Fedriani 2019).

Aggregated patterns in plants may be the rule rather than the exception (Condit et al. 2000; Shen et al. 2013; Wiegand et al. 2007, 2021; Wiegand, Martínez, and Huth 2009), and different aggregation mechanisms can underlie plant spatial distribution (Perry et al. 2013; Shen et al. 2013). Additionally, a plant spatial pattern may exhibit aggregation at different scales, whose relative importance may change with plant size, age, or species (Picard et al. 2009; Wiegand et al. 2007). Spatial point pattern analysis (SPPA) is a powerful tool that can identify the scales of plant aggregation and provide essential spatial information about the plant distribution (e.g., the density of clusters, average cluster size; Garrote, Castilla, and Fedriani 2019; Wiegand, Martínez, and Huth 2009; Wiegand and Moloney 2014). The main assumption of this type of analysis is that individual plants can be approximated as points (Wiegand and Moloney 2014).

Comparing the spatial distribution of a target plant species at different sites can help to identify potential underlying aggregation mechanisms (Getzin et al. 2008, 2015; Jácome‐Flores et al. 2016; Rodríguez‐Pérez, Wiegand, and Traveset 2012). For instance, Rodríguez‐Pérez, Wiegand, and Traveset (2012) compared the spatial distribution of *Daphne rodriguezii* in areas with and without *Podarcis lilfordi*, its sole seed disperser. They found that while plants were aggregated in all populations, those where the disperser was absent showed stronger aggregation at shorter distances. Perry et al. (2013) found that species in a fire‐prone shrubland communities that were killed by fire and recruited solely via seeds tended to have more aggregated distributions compared to species that survive fire and resprout.

Trees play a vital role in the maintenance of many ecosystems worldwide, while also providing multiple benefits to people (e.g., carbon storage, freshwater provisioning; Fajardo, McIntire, and Olson 2019; Lutz et al. 2018; Shvidenko, Barber, and Persson 2005). Unfortunately, a third of tree species on Earth are threatened with extinction, and their disappearance will lead to the loss of many other living organisms (Rivers et al. 2023). Under ongoing climate change, species with small populations, fragmented ranges, low fertility, or experiencing declines due to plagues and diseases appear to be more vulnerable than widespread species with large populations and high fecundity (Aitken et al. 2008). In this context, it is useful to characterize the spatial patterns of tree populations to provide insights into the ecological and historical factors contributing to such patterns and thus inform plant species conservation efforts.

In this study, we aim to determine the extent to which the spatial distribution pattern of the Iberian pear (*Pyrus bourgaeana*), a low‐density mammal‐dispersed tree, varies among local tree populations that show differences in the composition of their associated frugivores and herbivore assemblages. To this end, we applied spatial point pattern analysis to assess the spatial pattern of five fully mapped populations of the Iberian pear in southern Iberian Peninsula. Medium‐sized carnivores are the effective seed dispersers of Iberian pear, and overabundant wild ungulates act as seed and seedling predators, while small birds and rabbits act as pulp feeders that increase seedling emergence and thus local tree recruitment (Fedriani et al. 2024). Specifically, we tested two hypotheses: (i) because the clumped defecation behavior of their primary dispersers, such as the Eurasian badger ( *Meles meles* ), leads to a pattern of local aggregation, and because denser groups of fruiting trees can limit seed dispersal by attracting frugivores to specific sites, we hypothesize that all populations should show a consistent small‐scale aggregation pattern (Carlo and Morales 2008; Fedriani, Wiegand, and Delibes 2010). Additionally, populations with greater frugivore activity are expected to exhibit increased overall aggregation; and (ii) because ungulates reduce recruitment by preying on seeds and seedlings, we hypothesize that ungulate activity will show negative relationships with tree density and level of aggregation.

## 2 Materials and Methods

### 2.1 Study Area, Species, and Sites

The study was carried out in the Doñana Natural Space, which includes the Doñana National Park (high degree of protection) and Doñana Natural Park (lower degree of protection), located on the west bank of the Guadalquivir River estuary in southwest Spain (37°90 N, 6°260 W). Three main ecosystems can be found in the Doñana area: marshes, scrubland (where Iberian pear grows), and mobile dunes (Żywiec et al. 2017). The current characteristics of the Doñana ecosystems are the result of centuries of exploitation of natural resources, along with the complex interactions between plant and animal communities (Muñoz‐Reinoso, Jordán, and Tejada‐Tejada 2020). The climate is Mediterranean subhumid (537.7 ± 30.7 mm) characterized by dry (8.4 ± 1.4 mm), hot (10.7°C–38.5°C, minimum and maximum average temperatures) and long summers with extreme droughts (June–September). The mean minimum and maximum temperatures in winter are 0.1°C ± 0.3°C and 4.8°C ± 0.3°C. Most rainfall occurs during autumn (87.6 ± 7.6 mm) and winter (74.0 ± 7.3 mm) (Data from Natural Processes Monitoring Group, Doñana Biological Station).

**FIGURE 1:**
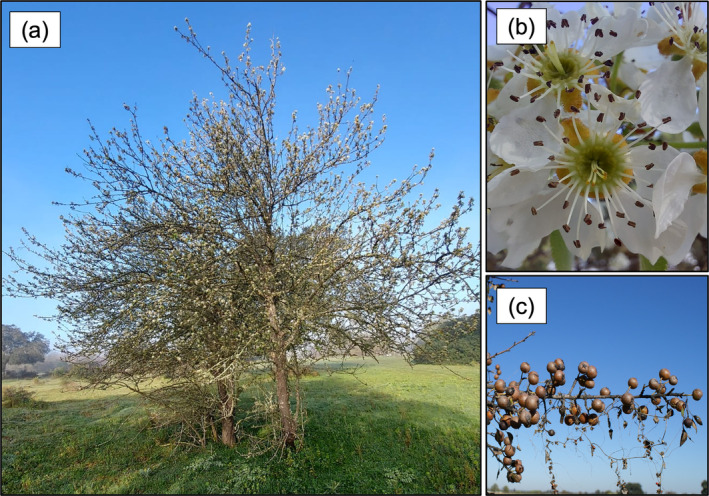
Study tree species Iberian pear (*Pyrus bourgaeana*) in the Doñana National Park (SW Spain). (a) adult tree, (b) flowers, and (c) infructescence with ripe fruits.

The Iberian pear (Figure 1) is a deciduous tree that belongs to the Rosaceae family, native to the Iberian Peninsula and northern Morocco, and a close relative of the domestic pear *Pyrus communis* (Aldasoro, Aedo, and Garmendia 1996). This small tree reaches heights of 3–6 m and flowers during February–March and usually produces 45–7500 fruits that ripen and fall to the ground during September–December (Fedriani and Delibes 2009a; Authors *pers. obs*.). Seeds are dispersed mainly by red foxes ( *Vulpes vulpes* ) and Eurasian badgers ( *Meles meles* ), which tend to deliver seeds in a contrasting way, relatively scattered or highly clumped, respectively (Fedriani, Wiegand, and Delibes 2010). Furthermore, while wild boar ( *Sus scrofa* ) occasionally disperses some seeds, red deer ( *Cervus elaphus* ) and fallow deer ( *Dama dama* ) act strictly as fruit and seed predators. Birds and rabbits operate primarily as pulp feeders, though corvids can also disperse some seeds (Fedriani et al. 2023, 2024). Seedlings usually emerge from late February to late April, and extensive seedling mortality occurs during their first summer due to droughts. Seedling emergence, survival, and recruitment success markedly vary among populations (Fedriani et al. 2019; Fedriani and Delibes 2009a). In the Doñana region, this tree exhibits low densities (usually < 1 individuals·ha^−1^) within several patches of Mediterranean scrub that are isolated from each other by unsuitable areas such as marshes, towns, or crops (Fedriani, Wiegand, and Delibes 2010). One‐third of saplings under reproductive trees have a probability greater than 0.9 of being clones though they seldom reach the adult stage (Castilla et al. 2019).

**FIGURE 2:**
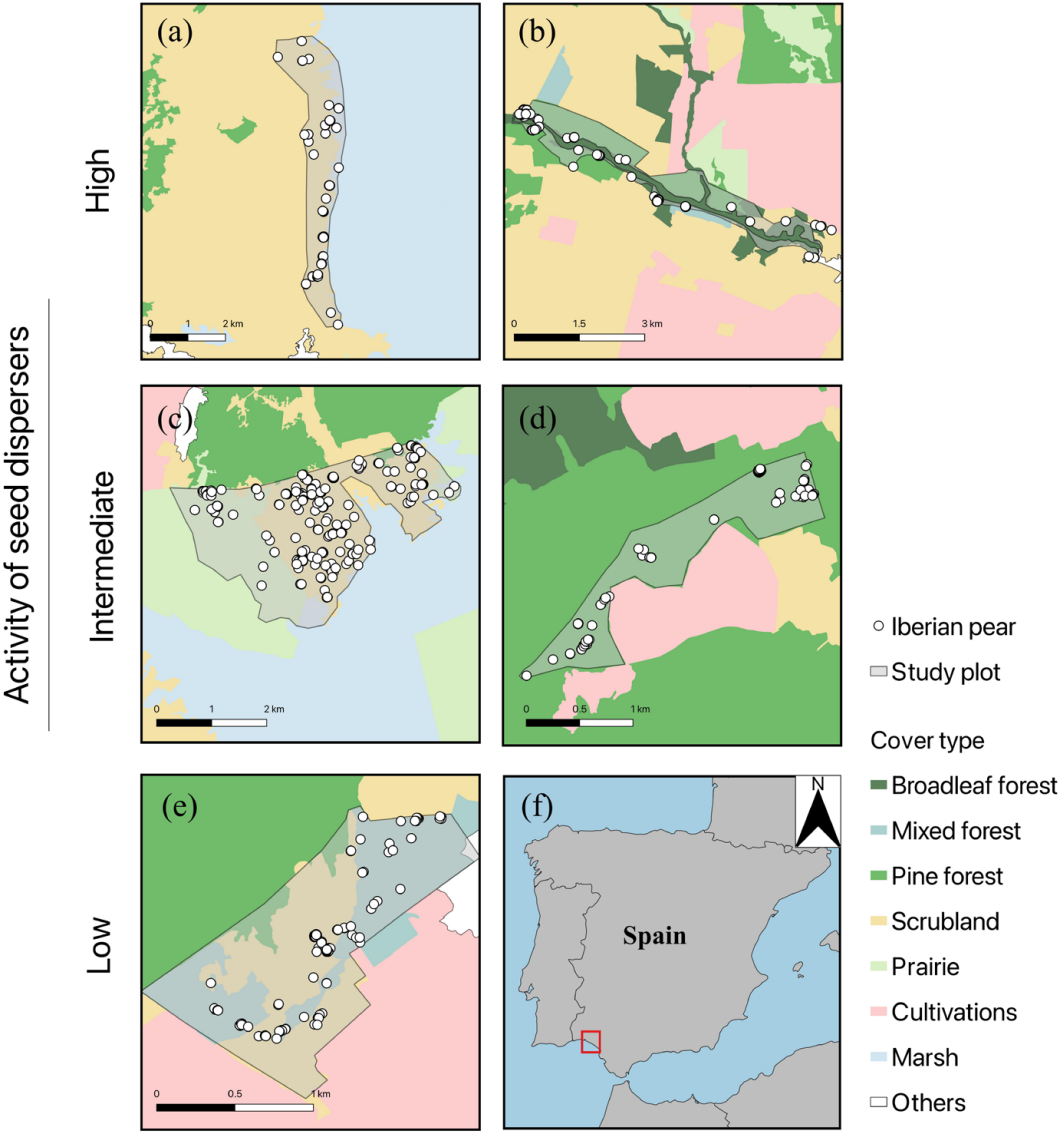
The study plots and Iberian pear trees (*Pyrus bourgaeana*, white dots) in the five study sites Reserva (a), Rocina (b), Matasgordas (c), Hinojos (d), and Hato Ratón (e) in the Doñana Natural Space in Southwest Spain (f).

We quantified the spatial distribution of five Iberian pear populations (called Hato Ratón, Hinojos, Matasgordas, Reserva, and Rocina) from 3.4 to 18.1 km apart (Figure 2). While all five sites are dominated by Mediterranean scrubland, there are variations in the activity of different frugivores and herbivores (Table 1). Matasgordas, Reserva, and Rocina are located within the National Park (high degree of protection), where hunting is prohibited, while Hato Ratón and Hinojos are located outside the National Park (lower degree of protection as Natural Park), where hunting is allowed. Hunting regulation leads to a much higher density of vertebrate herbivores and seed dispersers within the boundaries of the national park compared to outside and probably also leads to a higher activity (Garrote et al. 2018) (Table 1).

**TABLE 1:** The main characteristics of each study site in Doñana Natural Space where *Pyrus bourgaeana* was mapped.

Activity of seed dispersers	Site	Plot size (km^2^)	Density of trees (km^2^)	Degree of protection	Activity of seed predators
High	Reserva	6.81	18.65	High	High
	Rocina	4.19	30.78	High	Intermediate
Intermediate	Matasgordas	8.57	29.17	High	High
	Hinojos	1.39	64.74	Low	Low
Low	Hato Ratón	1.63	84.66	Low	Low

*Note:* Relative activity of seed dispersers and ungulate predators (visit likelihood: low = ≤ 0.2, intermediate = > 0.2 and ≤ 0.5, high = > 0.5; Garrote et al. 2018); degree of protection (high = within Doñana National Park, low = outside Doñana National Park).

### 2.2 Data Collection

To quantify the spatial distribution of Iberian pear trees at the five study sites, we delimited a sampling plot within each site (1.39–8.57 km^2^), making sure that each plot comprised at least 70 adult trees (minimum sample size needed for spatial analyses; Wiegand and Moloney 2014) and excluding flood‐prone areas that are not suitable for the Iberian pear (marshes and streams) (Figure 2a–e). All adult trees—reproductive or larger than 11 cm in circumference (the minimum circumference recorded in a reproductive individual)—within each plot were georeferenced using a submetric GPS (Leica 1200).

### 2.3 Spatial‐Point Pattern Analyses

To characterize the spatial distribution of Iberian pear in each sampling plot, we used univariate Thomas cluster point process models (see below) in the software *Programita* (Wiegand and Moloney 2014). *Programita* accounted for the irregular shape of the observation windows in our study plots by applying an edge correction (see Chapter 3.4.2.2 in Wiegand and Moloney 2014). For model selection, we employed four summary functions that are known to provide a comprehensive description of the spatial structure of homogeneous patterns: (i) the pair correlation function *g*(*r*), (ii) the *L*‐function *L*(*r*), (iii) the spherical contact distribution *H*_*S*_(*r*), and (iv) the nearest‐neighbor distribution function *D*(*r*) (Illian et al. 2008; Wiegand, He, and Hubbell 2013; Wiegand and Moloney 2014).

We used the second‐order summary functions *g*(*r*) and *L*(*r*) to fit the parameters of the Thomas processes, as these functions are particularly sensitive to aggregation at small and larger scales, respectively; the joined use of these two functions allowed us to improve the parameter fitting (Wiegand, Martínez, and Huth 2009). The *g*(*r*) measures spatial aggregation of a species in a study plot by comparing the mean neighborhood density of Iberian pear a distance *r* away with the average density in the plot; thus, a value of *g*(*r*) = 1 indicates randomness, while *g*(*r*) > 1 indicates spatial aggregation with a density of the neighborhood higher than expected by a random pattern. The *L*(*r*) measures the degree of aggregation or dispersion of a species within a study plot by comparing the total number of neighbors within distance *r* to the expected number under a random pattern; a value of *L*(*r*) > 0 indicates aggregation, while *L*(*r*) < 0 indicates hyperdispersion (regularity).

However, the functions *g*(*r*) and *L*(*r*) are often insufficient to characterize more complex spatial patterns that may contain gaps, isolated points, or areas of low point density (Wiegand, He, and Hubbell 2013). Therefore, we supplemented the analysis with the nearest‐neighbor summary functions *H*_*S*_(*r*) and *D(r)*. *H*_*S*_(*r*) characterizes the size of “holes” in the pattern by indicating the proportion of random test locations that have at least one neighbor of the pattern within the distance *r*. Meanwhile, *D*(*r*) characterizes small‐scale aggregation by revealing the probability that plants have at least one neighbor within distance *r* (Illian et al. 2008; Wiegand, He, and Hubbell 2013).

#### 2.3.1 Description of Thomas Process Models

For each sampling plot, we fitted the following four Thomas processes (Wiegand, Martínez, and Huth 2009; Wiegand and Moloney 2014) to the spatial distribution patterns of the Iberian pear in our five study plots:

1. Single‐cluster process: The Thomas process with one critical scale of clustering is a stochastic model that involves a random and independent distribution of clusters in a given area *A*. The intensity of the cluster centers, or the number of clusters per unit of area, is denoted by *ρ*. Points within the pattern are randomly assigned to these clusters, and the distances of the points relative to the center of the cluster follow a two‐dimensional normal distribution with variance *σ* ^2^. The size of the resulting cluster *r*_C_ is approximately twice the standard deviation of the normal distribution (*r*_C_ ≈ 2*σ*) and covers approximately 87% of the points within a given cluster. The area covered by one cluster (*A*_*C*_) is then equal to *π r*_C_^2^ or 4*πσ* ^2^. The pair correlation function of this Thomas process is:

(1) $$g\left(r,\sigma ,\rho \right)=1+\frac{1}{\rho }\frac{\exp\left(-r^{2}/4\sigma^{2}\right)}{4\pi \sigma^{2}}$$

2 Generalized single‐cluster process: The second point process utilized in this study is a Thomas process with one critical scale of clustering that allows for a clumped distribution of the number of points assigned to the clusters, meaning that many clusters host few individuals, whereas few clusters host many individuals. This can be accomplished by using a negative binomial distribution with clumping parameter *k* (Wiegand and Moloney 2014). Note that the two single‐cluster processes cannot be distinguished based on the *g* or *L* functions alone. However, the generalized single‐cluster process (2) is required if fitting the single‐cluster process (1) to the observed pattern shows that the spherical contact distribution *H*_*S*_(*r*) is clearly underestimated and the distribution function of the distances to the nearest‐neighbor *D*(*r*) is overestimated. In this case, the observed pattern has a higher heterogeneity in the number of points per cluster (e.g., more isolated points) than the pattern generated by the single‐cluster process (1). The pair correlation function of this Thomas process is:

(2) $$g\left(r,\sigma ,\rho \right)=1+\frac{f_{k}}{\rho }\frac{\exp\left(-r^{2}/4\sigma^{2}\right)}{4\pi \sigma^{2}}$$

where the factor *f*_*k*_ = (1 + *k*)/*k*. For large values of *k*, the generalized single‐cluster process reduces to the single‐cluster process since *f*_*k*_ ≈ 1. To determine the value of *k*, we repeated the fitting procedure for several values of *k* and evaluated the fit of *H*_*S*_(*r*) and *D*(*r*).

3 Double‐cluster process: The Thomas process with two critical scales follows the same principles as the model (1) with one critical scale of clustering. The key difference is that the cluster centers in this model do not follow a random distribution, but instead conform to a Thomas process (1) with one critical scale of clustering. Thus, this process produces a spatial structure where small clusters are nested within large clusters. This “double‐cluster” process has four unknown parameters: the intensities *ρ*_*L*_ and *ρ*_*S*_ of the large and small cluster centers, respectively, as well as the parameters *σ*_*L*_ and *σ*_*S*_, which specify the size of the large and small clusters, respectively. The pair correlation function of this Thomas process is:

(3) $$g\left(r,\sigma ,\rho \right)=1+\frac{1}{\rho_{S}}\frac{\exp\left(-r^{2}/{4\sigma }_{S}^{2}\right)}{4{\pi \sigma }^{2}}+\frac{1}{\rho_{L}}\frac{\exp\left(-r^{2}/4\left(\sigma_{S}^{2}+\sigma_{L}^{2}\right)\right)}{4\pi \left(\sigma_{s}^{2}+\sigma_{L}^{2}\right)}$$

4 Generalized double cluster: The fourth‐point process utilized in this study is a Thomas process with two critical scales of clustering, which allows in analog to process (2) for a clumped distribution of points among the small clusters and a clumped distribution of small clusters among the large clusters (Wiegand and Moloney 2014). Again, the two double‐cluster processes cannot be distinguished based on the *g*‐ or *L*‐function, but by using additionally the spherical contact distribution *H*_*S*_(*r*) and the nearest‐neighbor distribution function *D*(*r*). The pair correlation function of this Thomas process is:

(4) $$g\left(r,\sigma ,\rho \right)=1+\frac{f_{\mathrm{kS}}}{\rho_{S}}\frac{\exp\left(-r^{2}/{4\sigma }_{S}^{2}\right)}{4{\pi \sigma }^{2}}+\frac{f_{\mathrm{kL}}}{\rho_{L}}\frac{\exp\left(-r^{2}/4\left(\sigma_{S}^{2}+\sigma_{L}^{2}\right)\right)}{4\pi \left(\sigma_{s}^{2}+\sigma_{L}^{2}\right)}$$

Note the two additional factors *f*_ks_ and *f*_kl_ in process (4), which account for the clustering of the negative binominal distribution, with *f*_*k*_ = (*k* + 1)/*k*. Thus, for *k* → ∞ we have *f*_*k*_ → 1 so we recover the simpler process (3). To determine the values of clumping parameters *k*, we fitted the process for different parameter values of the clumping parameters *k* for small and/or large clusters, for example, *k* = 0.1 (i.e., *f*_*kL*_ = 11 and *f*_*kS*_ = 11).

We selected the simplest point process that fitted the four summary functions simultaneously, by testing their agreement with the observed summary functions by conducting 199 simulations of the fitted point processes and estimating pointwise simulation envelopes with an approximate error rate of *α* = 0.05 (Wiegand and Moloney 2014). The simulation envelopes were defined as the fifth lowest and highest values of the summary functions of the simulated point process. To test the overall departure of the data from the null model (i.e., each point process) without type I error inflation, we used a goodness‐of‐fit (GoF) test (Loosmore and Ford 2006) that collapses the scale‐dependent information contained in the test statistics into a single test statistic *u*_*i*_ which represents the total squared deviation between the observed pattern and the theoretical result across the scales of interest (i.e., 0–100 m). The *u*_*i*_ is calculated for the observed data (*i* = 0) and for the data created by *i* = 1, …, 199 simulations of the null model and the rank of *u*_0_ among all *u*_*i*_ is then determined. If the rank of *u*_0_ is > 190, there is a significant departure from the null model with *α* = 0.05 on the scales of interest. Thus, the model with no significant differences (*p*‐value ≥ 0.05) from our data in all summary functions is the one that best explains the observed spatial pattern.

The Thomas process models provided us with a set of up to eight parameters that can be used to characterize the distribution of Iberian pear within a sampling plot. These parameters are as follows: the density of large clusters (*ρ*_L_), the density of small clusters (*ρ*_S_), the ratio of the number of small clusters to the number of large clusters (*ρ*_S_/*ρ*_L_), the size of large clusters (2*σ*_L_ m), the size of small clusters (2*σ*_S_ m), the ratio of the size of large clusters to the size of small clusters (*σ*_L_/*σ*_S_), the mean number of individuals in a large cluster (*μ*_L_), and the mean number of individuals in a small cluster (*μ*_S_). We adjusted the estimated number of clusters based on the plot area to allow for valid comparisons between sites.

To analyze the relationships between ungulate activity and various tree distribution parameters, we conducted a Kendall's *τ* correlation analysis. Ungulate activity was ranked across the five populations, along with the parameters that characterize the distribution of Iberian pear. Each parameter was assigned a rank from 1 to 5, with 1 indicating the population with the lowest value and 5 the highest. This ranking approach allowed us to assess trends and potential associations across our study populations.

## 3 Results

### 3.1 Overall Patterns

A total of 734 Iberian pear trees were georeferenced in the five study plots (range 90–250). The average density was 45.6 ± 12.4 trees·km^2^ (mean ± SE), with the highest density observed in Hato Ratón (84.6 trees·km^2^), followed by Hinojos (64.7 trees·km^2^), Rocina (30.7 trees·km^2^), Matasgordas (29.1 trees·km^2^), and Reserva (18.6 trees·km^2^).

**FIGURE 3:**
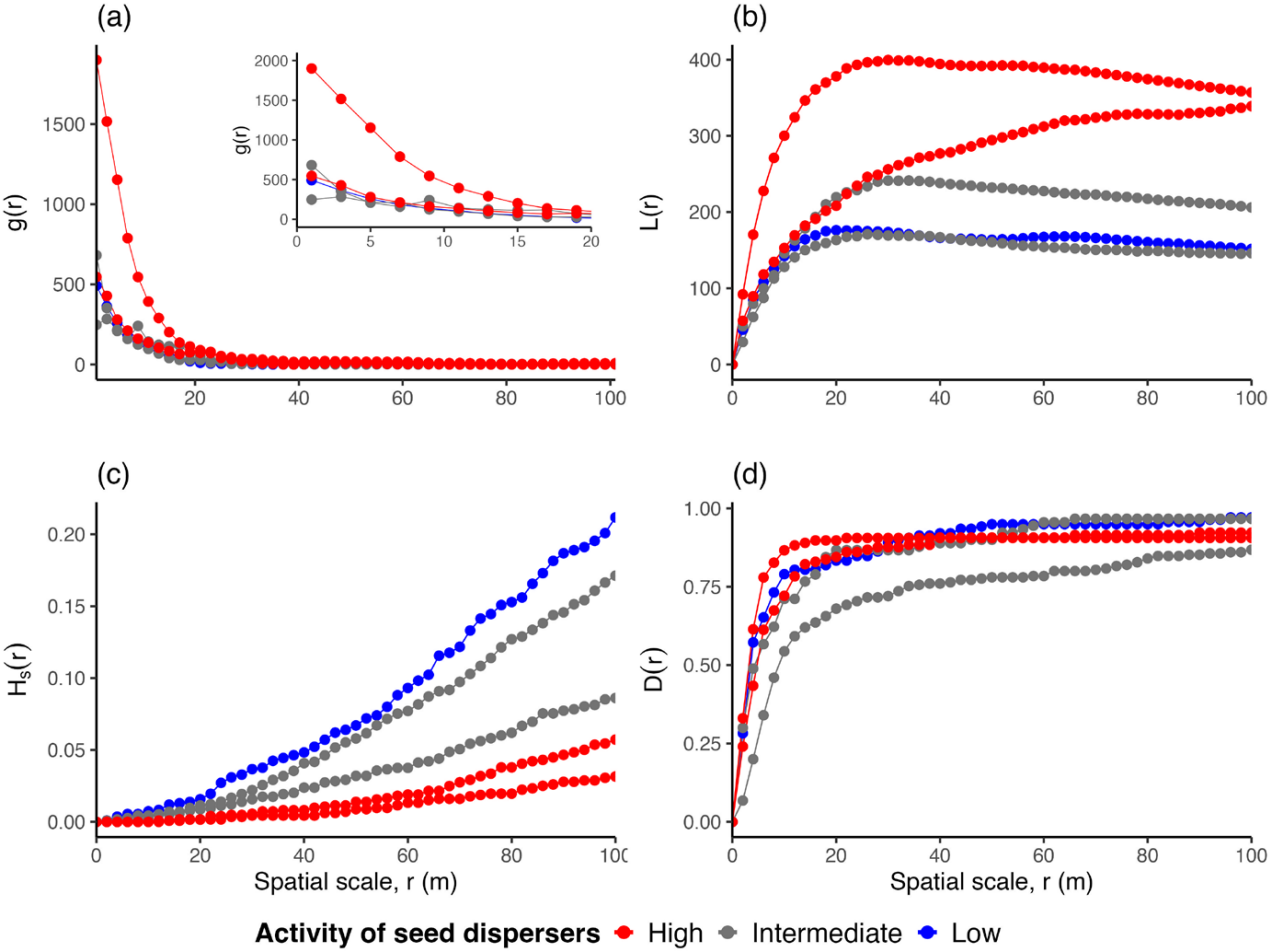
Observed summary functions describing the spatial distribution of adult Iberian pear (*Pyrus bourgaeana*) in five study sites with different activity of seed dispersers. Shown are the pair correlation function *g*(*r*) (a), the *L*‐function *L*(*r*) (b), the spherical contact distribution *Hs*(*r*) (c), and the nearest‐neighbor distribution function *D*(*r*) (d).

**FIGURE 4:**
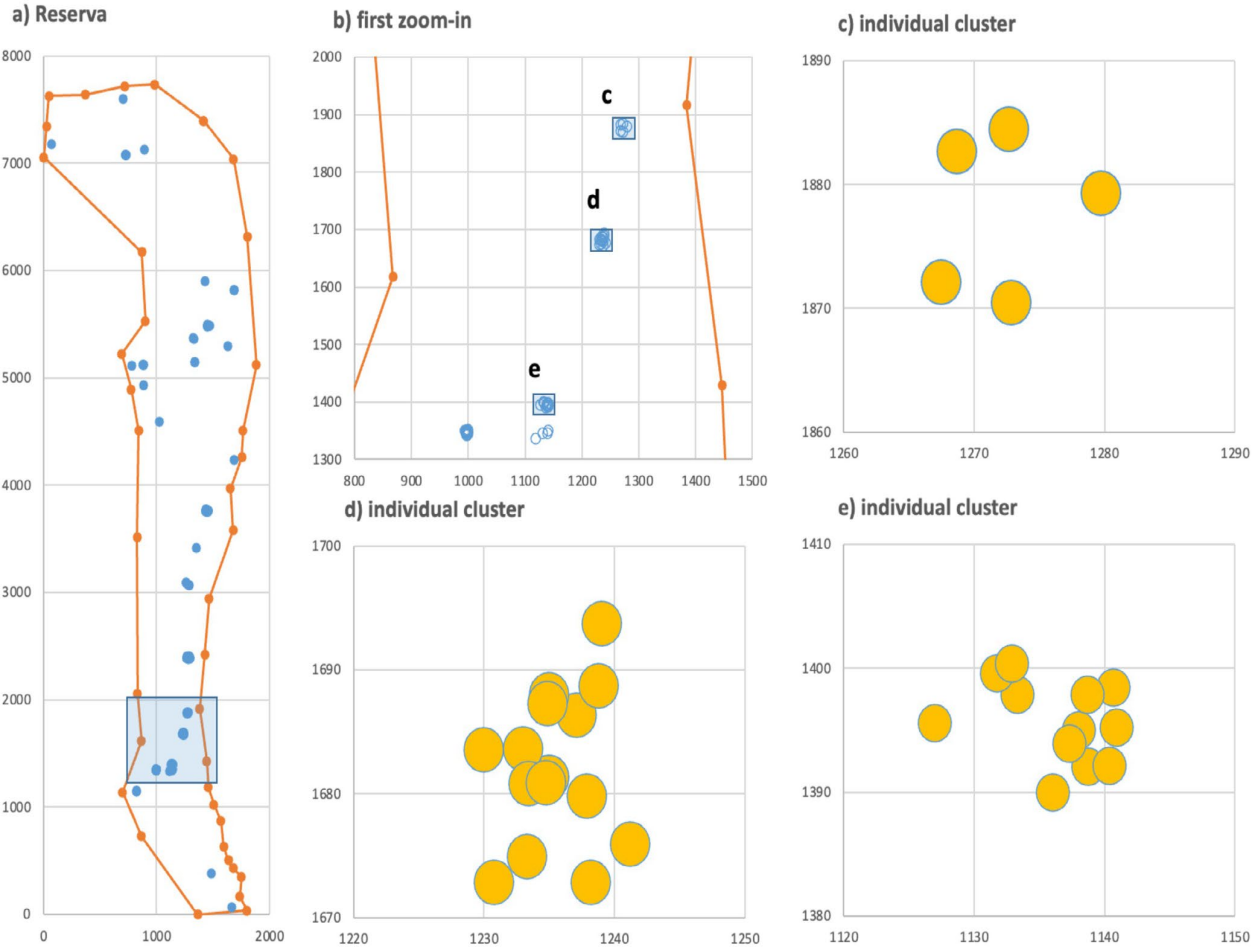
Illustration of the scaling of aggregation for the 90 reproductive Iberian pear trees at the Reserva plot. While clusters are distributed within the study areas over several km (a), usually several hundred meters away of each other (b), the size of individual clusters ranges at scales of tens of meters (c—e). This particular spacing leads to very high levels of aggregation. Distances are given in m.

In all plots, the pair correlation function *g*(*r*) exhibited strong small‐scale aggregation, with neighborhood densities being 124 to 1900 times higher than expected under complete spatial randomness. Note that *g*(*r*) > 1 indicates spatial aggregation with a higher density of neighbors than expected by a random pattern (Figure 3a). The extremely high aggregation of the patterns of Iberian pear trees results from an approximately random distribution of clusters over a large area (several km^2^), with aggregation occurring at scales of tens of meters (Figure 4). Interestingly, the pair correlation functions were very similar among populations, except that the Reserva plot showed a higher degree of small‐scale spatial aggregation. For example, the aggregation level at 5 m in Reserva, a population characterized by high seed disperser activity (Table 1), was 5.5 times greater than that in Hinojos and Matasgordas (intermediate disperser activity), 4.4 times higher than in Hato Ratón (low activity), and 4.1 times higher than in Rocina, another site with high disperser activity (Figure 3a). Similarly, the *L*‐functions of all plots, except Reserva, exhibited consistent patterns beyond 20 m, with the highest values in sites with high activity of seed dispersers and the lowest in the sites with low and intermediate activity of seed dispersers (Figure 3b).

The *H*_*s*_(*r*) function, the probability that the next Iberian pear individual was within distance *r* of a random location in the study plot, showed that the smallest gaps were observed in a plot with low activity of seed dispersers (e.g., approximately a 21% probability for *r* = 100 m at the Hato Ratón plot), intermediate gaps were observed in plots with intermediate activity of dispersers (approximately 11% and 12% at the Hinojos and Matasgordas plot), and large gaps were observed in the plots with high activity of seed dispersers (approximately 6% and 3% at the Reserva and Rocina plot; Figure 3c). Furthermore, the nearest‐neighbor distribution function *D*(*r*) was very similar in all cases except for the plot with intermediate activity of seed dispersers (Matasgordas), which was lower at all scales (Figure 3d). Note that the *D*(*r*) indicates that approximately 75% and 90% of all individuals in Matasgordas and all other plots had their nearest neighbor within 40 m (Figure 3d), which illustrates the high level of small‐scale aggregation.

### 3.2 Cluster Analyses

The fit of the single‐cluster Thomas process (1) was unsatisfactory; the *H*_*s*_(*r*) and *D*(*r*) functions of the fitted point process strongly and consistently deviated in all five sites from the observed summary functions (Table 2). Specifically, this point process underestimated the size of the gaps in the patterns and predicted that almost all Iberian pear individuals would have their nearest neighbor within some 30 m (i.e., inside the cluster) (Appendix 1), whereas the observed data indicated a substantial proportion of single individual clusters. The observed pair correlation function *g*(*r*) showed some weaker departures from the simulation envelopes (Table 2). Indeed, the generalized single‐cluster Thomas process (2), which allows for variability in the number of individuals per cluster (and for a higher proportion of single individual clusters), showed a good fit for all summary functions at all sites, except for the *D*(*r*) function in Rocina (Table 2), although departures from the simulation envelopes therein were small (Appendix 1).

**TABLE 2:** Summary of the goodness‐of‐fit (GoF) test of different Thomas cluster point process models for the four summary functions describing the spatial distribution of adult pear trees (*Pyrus bourgaeana*) in each study site.

Activity of seed dispersers	Site	*N*	Thomas process	Summary functions							
				*g*(*r*)		*L*(*r*)		*H*_S_(*r*)		*D*(*r*)	
				Rank	*p*‐value	Rank	*p*‐value	Rank	*p*‐value	Rank	*p*‐value
High	Reserva	127	SC	**191**	**0.05**	175	0.13	**200**	**0.005**	**200**	**0.005**
			GSC (*k* = 0.1)	92	0.54	38	0.81	95	0.53	141	0.30
			DC	78	0.61	15	0.93	**199**	**0.01**	**200**	**0.005**
			GDC (*k*_L_ = 0.1, *k*_s_ = 0.1)	19	0.91	31	0.85	36	0.82	189	0.06
	Rocina	129	SC	**200**	**0.005**	**191**	**0.05**	**200**	**0.005**	**200**	**0.005**
			GSC (*k* = 0.1)	179	0.11	46	0.77	68	0.66	**192**	**0.04**
			DC	**195**	**0.03**	181	0.1	**194**	**0.03**	**200**	**0.005**
			GDC (*k*_L_ = 0.1, *k*_s_ = 0.1)	142	0.29	12	0.94	92	0.59	**192**	**0.04**
Intermediated	Matasgordas	250	SC	147	0.27	129	0.36	**200**	**0.005**	**200**	**0.005**
			GSC (*k* = 0.1)	82	0.59	37	0.82	83	0.59	185	0.08
			DC	137	0.32	80	0.60	**200**	**0.005**	**200**	**0.005**
			GDC (*k*_L_ = 9999, *k*_s_ = 0.1)	120	0.40	67	0.67	**200**	**0.005**	**200**	**0.005**
	Hinojos	90	SC	183	0.09	180	0.10	**200**	**0.005**	**200**	**0.005**
			GSC (*k* = 0.1)	102	0.49	60	0.70	45	0.78	147	0.27
			DC	188	0.06	168	0.16	**200**	**0.005**	**200**	**0.005**
			GDC (*k*_L_ = 9999, *k*_s_ = 0.1)	184	0.08	155	0.23	**200**	**0.005**	**200**	**0.005**
Low	Hato Ratón	138	SC	**194**	**0.03**	168	0.16	**200**	**0.005**	**200**	**0.05**
			GSC (*k* = 0.1)	57	0.72	26	0.87	61	0.70	179	0.11
			DC	186	0.07	115	0.13	**200**	**0.005**	**200**	**0.005**
			GDC (*k*_L_ = 0.1, *k*_s_ = 0.1)	37	0.82	8	0.96	145	0.28	189	0.06

*Note:* The Thomas process models (SC: Single‐clustered; GSC: Generalized single‐clustered; DC: Double‐clustered; GDC: Generalized double‐clustered) were fitted to the observed pair correlation and *L*‐functions. *N* = number of adult trees. *g*(*r*) = pair correlation function; *L*(*r*) = *L‐function*; *H*_S_(*r*) = spherical contact distribution; *D*(*r*) = nearest‐neighbor distribution function; *k* = distribution of the individuals over the clusters in the negative binomial distribution. In bold significant differences between the fitted point process model and the observed values.

The Thomas process (3) with two critical scales of clustering (double cluster) suffered from the same problem as the single‐cluster Thomas process (1), as it underestimated the number of single individual clusters, which caused strong departures from the observed *Hs*(*r*) and *D*(*r*) functions (Table 2; Appendix 1). Finally, the generalized double‐cluster Thomas process (4) exhibited a good fit for all summary functions at all sites, except for the *H*_*s*_(*r*) function in Hinojos and Matasgordas and *D*(*r*) function in Hinojos, Matasgordas, and Rocina (Table 2), although departures from the simulation envelopes were small (Appendix 1). To sum up, both the generalized single‐cluster and generalized double‐cluster Thomas process exhibited a similar fit, but we selected the more parsimonious generalized single‐cluster point process.

The analyses for the best fitted generalized single‐cluster Thomas processes revealed an average number of 4.4 ± 1.2 clusters per km^2^, a cluster radius of 8.5 ± 1.8 m, and the highest cluster radius was observed in Rocina, followed by Hinojos, Matasgordas, Hato Ratón, and Reserva (Table 3). On average, these clusters encompassed 11.7 ± 2.2 trees. The number of trees per cluster in the Hinojos plot was 4.3, 1.5, 1.3, and 1.2 times higher than in the Matasgordas, Reserva, Hato Ratón, and Rocina plots, respectively (Table 3).

**TABLE 3:** Summary of the results of the *Pyrus bourgaeana* spatial distribution fitted with the best‐adjusted Thomas cluster process.

Activity of seed dispersers	Site	Density of trees (km^2^)	*ρ* (km^2^)	2*σ* (m)	*μ*
High	Reserva	18.65	1.67	5.77	11.11
	Rocina	30.78	2.15	15.5	14.28
Intermediate	Matasgordas	29.17	7.58	6.48	3.85
	Hinojos	64.74	3.88	9.03	16.67
Low	Hato Ratón	84.66	6.77	5.88	12.50

*Note: ρ* = the number of clusters per km^2^; 2*σ* (m) = the size of clusters; *μ* = average number of individuals in one cluster.

**FIGURE 5:**
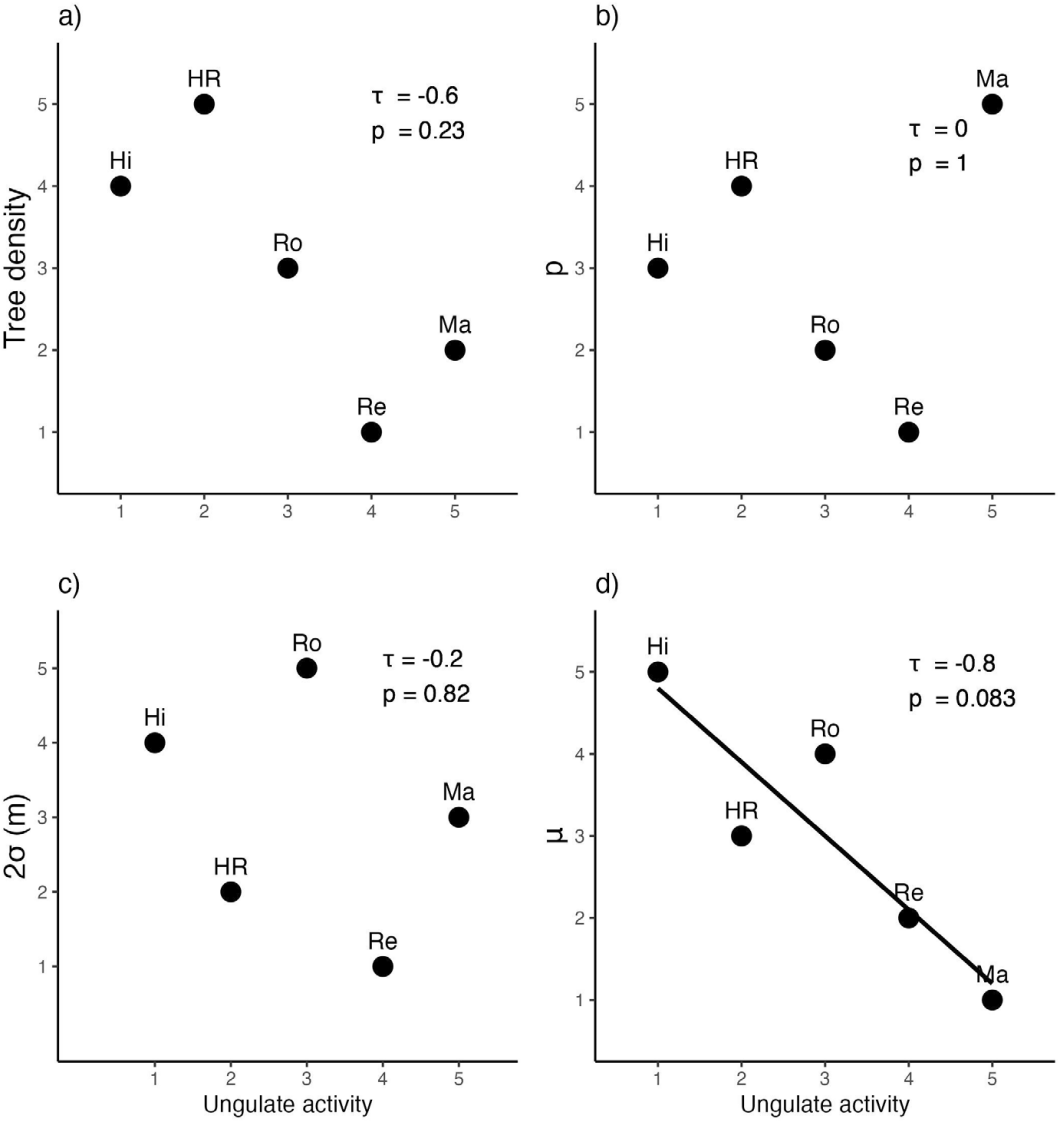
Kendall rank correlation analysis between ranked ungulate activity and ranked tree distribution parameters: (a) tree density, (b) density of clusters (*ρ* km^2^), (c) radius of cluster (2*σ* (m)), and (d) number of trees per cluster (*μ*), showing *τ* and *p*‐values for each correlation. The acronyms of the points show the name of the study plot: Hato Ratón (HR), Hinojos (Hi), Matasgordas (Ma), Reserva (Re), and Rocina (Ro).

No significant correlations were found between ungulate activity and the overall tree density (Figure 5a). However, there was a noticeable trend toward lower tree density in areas with higher ungulate activity. Also, a marginally significant negative correlation was detected between ungulate activity and the number of trees per cluster (Figure 5d), suggesting that in areas with higher ungulate activity, trees tend to form less dense clusters. Moreover, cluster density did not show a clear relationship with ungulate activity (Figure 5b) and cluster size (Figure 5c).

## 4 Discussion

Evaluating the spatial distribution of plants is crucial for understanding ecological processes in community composition and dynamics (Ben‐Said 2021; Law et al. 2009; Velázquez et al. 2016). Notably, our spatially explicit approach revealed largely consistent distribution patterns in five Iberian pear populations, characterized by strong small‐scale aggregation, a low density of clusters, and high variability in the number of trees per cluster. The only exception was the Reserva plot that showed high activity of seed dispersers and substantially higher aggregation than the other plots. This result supports partially our first prediction that populations with high seed dispersers activity would show higher aggregation, and suggests that the denser groups of fruiting trees can limit seed dispersal distance by attracting frugivores, helping to create and maintain plant aggregation (Carlo and Morales 2008; Fedriani, Wiegand, and Delibes 2010). Furthermore, the results do not provide support for our second prediction that ungulate activity would be negatively correlated with tree the density and level of aggregation, potentially reducing recruitment by preying on seeds and seedlings (Fedriani et al. 2024).

Our results are consistent with those previously reported for the Iberian pear in one locality (Fedriani, Wiegand, and Delibes 2010; Garrote, Castilla, and Fedriani 2019). In both previous studies and throughout our study plots, the Iberian pear showed an aggregated distribution at distances of less than 25 m, with a single‐cluster scale of aggregation and a very low density of clusters. Furthermore, the low density and highly aggregated distribution observed in the Doñana region is also consistent with patterns recorded in other regions of southern Spain (Arenas‐Castro, Fernández‐Haeger, and Jordano‐Barbudo 2016; Burgos et al. 2022; Authors pers. obs.). However, this pattern may differ in other related species, for example, the Callery pear ( *Pyrus calleryana* ), an invasive bird‐dispersed species in the United States, has been found to show a random distribution of adult trees likely due to the species shade intolerance (Boyce 2024).

Plant populations showing simultaneously short‐ and long‐distance dispersal mechanisms often exhibit complex patterns of spatial distribution (e.g., several critical scales of clustering) (Howe 1989; Wiegand, Martínez, and Huth 2009). Given that Iberian pear shows long‐ and short‐distance seed dispersal by carnivores and clonal propagation as well (Castilla et al. 2019; Fedriani et al. 2023), a spatial pattern with several critical scales of clustering was expected. Surprisingly, we observed a consistent, simplified, and highly aggregated pattern with only one critical clustering scale, but high variability in the number of trees per cluster. The consistency in the spatial patterns among populations points to a common set of mechanisms that drive the overall distribution pattern, which may then vary due to local conditions.

Technically, the aggregation at a small scale, as measured by the pair correlation function, is determined by the quantity $1/\left(\rho \times \pi {\left(2\sigma \right)}^{2}\right)$ (Equations 1 and 2), where ρ is the density of clusters and 2σ the approximate radius of the clusters. Thus, small‐scale clustering may stay unchanged if a larger number of clusters are compensated by smaller clusters. This is what happens with the patterns of our five Iberian pear populations, which showed similar degrees of aggregation but differences in the size and the density of clusters (Table 3). However, the pattern at Reserva stands out by showing both a small cluster size and a low number of clusters, which results in higher small‐scale aggregation (Figure 3a).

An additional characteristic of the patterns of the five populations is their high variability in the number of individuals per cluster. A substantial proportion of Iberian pear individuals had their nearest neighbor at distances > 100 m (Reserva: 9%, Rocina: 8%, Matasgordas: 16%, Hinojos: 3%, Hato Raton: 3%; Appendix 1; Figure 3c), which is clearly outside their “home cluster” (the variability of these values is largely driven by the tree density). This suggests that the species shows typically a certain proportion of clusters with few individuals and a few clusters with a higher number of individuals. Such a pattern may occur if local environmental conditions vary or if the population exhibits very limited reproductive and regenerative capacity. In the following, we discus mechanisms that may lead to the observed strong small‐scale aggregation.

### 4.1 Ecological Processes Affecting Tree Spatial Distribution

Seed dispersal and predation are key mechanisms that affect patterns of regeneration and spatial structure in plant communities (Klinger and Rejmánek 2010, 2013; Orrock et al. 2006). The seedling spatial distribution may be restricted by low rates of seed dispersal and high seed predation rates (Cordeiro et al. 2009; Klinger and Rejmánek 2010; Silman, Terborgh, and Kiltie 2003). In the Doñana area, ungulates consume a high proportion of Iberian pear fruits, while only a small percentage are consumed by seed‐dispersing carnivores (Selwyn et al. 2020; Fedriani et al. 2024). Therefore, ungulate seed predators probably limit seed availability by preying on most seeds. Consequently, we found that the two sites with low ungulate activity (Hinojos and Hato Raton) showed a two to five time higher density of trees compared to sites with intermediate to high activity of ungulates (Table 3; Figure 5).

Limited dispersal can produce strong spatial associations of seeds with adults, resulting in spatial patterns that can persist beyond the seedling stage. In the Doñana area, seed dispersers often deposit their feces in close proximity to adult Iberian pears (Fedriani, Wiegand, and Delibes 2010). However, adult proximity also may lead to competition and density‐dependent mortality that reduces aggregation in seedlings (Condit et al. 2000; Rodríguez‐Pérez, Wiegand, and Traveset 2012). This highlights the importance of other underlying ecological processes in the aggregated recruitment of Iberian pear. For instance, clonal species have generally more aggregated patterns than nonclonal species. About one‐third of Iberian pear saplings are clonal shoots instead of recruits from sexual reproduction (within a 10‐m radius of adult trees), but only a small portion of these putative clonal shoots typically reaches sexual maturity (Castilla et al. 2019). Besides, Iberian pear pulp feeders (birds, rabbits) enhance local recruitment by pericarp removal, improving seed performance and seedling fate, leading to higher recruitment underneath mother trees (Fedriani, Zywiec, and Delibes 2012). These mechanisms could substantially contribute to the high small‐scale aggregation observed in adult Iberian pear trees.

Another ecological process involved in tree spatial aggregation is the clumped fecal making behavior by seed dispersers (Fragoso, Silvius, and Correa 2003; González‐Zamora et al. 2014; Kwit, Levey, and Greenberg 2004). In the case of the Iberian pear, one of its main seed dispersers (Fedriani et al. 2024), the Eurasian badger, delivers feces and seeds in a highly clumped pattern, creating the initial template, at least partially, for Iberian pear aggregation (Fedriani, Wiegand, and Delibes 2010; Fedriani and Delibes 2009a; Garrote, Castilla, and Fedriani 2022). Furthermore, most of the seed dispersal events and recruitment of Iberian pear take place over intermediate distances (250–1000 m) from the mother tree (Fedriani et al. 2023). Given that we measured aggregation at a scale of 0–100 m, the clumped defecation behavior of the main seed dispersers, along with their tendency to transport seeds over intermediate distances, could contribute to high tree aggregations at smaller scales (< 15 m).

The tree spatial pattern can also be affected by several postdispersal processes related to habitat suitability, seedlings and saplings predation, and biotic interactions. For example, Iberian pear exhibits extensive mortality of seedlings due to summer droughts and fungal infection (Fedriani and Delibes 2009b). Moreover, seedling emergence, survival, and recruitment success markedly vary across populations (Fedriani et al. 2019), adding an additional layer of complexity. Also, ungulates depredate on Iberian pear seedlings and saplings (Castilla et al. 2019). Furthermore, a positive spatial association has been observed among Iberian pear recruitment and the presence of some nurse species (e.g., *C. humilis* , *Halimium halimifolium*, *Stauracanthus genistoides*; Garrote, Castilla, and Fedriani 2019, 2021). Thus, postdispersal processes and variability in nurse species density could lead to the quantitative nuances observed in the spatial patterns of different populations.

### 4.2 Outcomes of Strong Tree Clustering

Plant spatial patterns are the template for future ecological and evolutionary dynamics (Law et al. 2009; Velázquez et al. 2016; Wiegand, Martínez, and Huth 2009; Wiegand et al. 2021). The aggregated pattern of individuals of Iberian pear has significant implications for the reproductive success of the species, management strategies, and ultimately the long‐term persistence of populations. For example, aggregated individuals receive fewer pollinator visits and have smaller crop sizes due to intraspecific competition for resources (Alonso‐López, Garrote, and Fedriani 2022). They are visited more frequently by some seed dispersers, but seed dispersal distances decrease with higher tree aggregation (Carlo and Morales 2008; Pegman, Perry, and Clout 2017; Fedriani et al. 2018). High conspecific density can lead to increased seed predator and pathogen encounters (Janzen 1970; Kolb, Leimu, and Ehrlén 2007; Mezquida and Olano 2013) and may promote predator satiation, reducing per capita predation chances (Augspurger 1981; but see Bogdziewicz et al. 2018). These findings underscore the critical role of tree distribution in population persistence. Finally, recent simulation research suggests that the way in which Iberian pear trees are distributed can profoundly affect seed arrival rates in target areas. Specifically, planting trees in an aggregated fashion has been shown to yield a more restricted seed rain compared to regular or random planting schemes (Fedriani et al. 2018). This emphasizes the need to carefully deliberate tree distribution strategies in specie management.

## 5 Conclusion

Despite the fact that the spatial distribution pattern of the Iberian pear is influenced by a complex interplay of several ecological processes, the spatial patterns of our five populations showed a high structural similarity. Determining the precise contribution of each of these processes to spatial patterning represents a significant challenge. In the Doñana Natural Space, the prevalence of ungulate‐dominated habitats imposes constraints on seed dispersal for Iberian pear, which can pose a significant challenge to its long‐term viability. However, the capacity for clonal propagation within the species may serve as a mechanism that fosters population persistence, particularly during unfavorable environmental conditions (e.g., severe summer droughts). Promoting the protection of clones growing next to old and/or dead trees is a management strategy to assist the medium‐term viability of populations. Monitoring spatial distribution patterns across various populations can enhance our ecological understanding of plant species, thereby facilitating a more comprehensive approach to assessing its conservation status. This holistic understanding is crucial to devise effective conservation strategies and ensure sustainable management of species.

## Author Contributions

**Brayan Morera:** conceptualization (equal), data curation (lead), formal analysis (lead), investigation (equal), methodology (equal), visualization (lead), writing – original draft (lead), writing – review and editing (lead). **Pedro J. Garrote:** conceptualization (equal), formal analysis (supporting), methodology (supporting), writing – original draft (supporting), writing – review and editing (supporting). **Thorsten Wiegand:** conceptualization (supporting), formal analysis (supporting), writing – original draft (supporting), writing – review and editing (supporting). **Daniel Ayllón:** conceptualization (supporting), formal analysis (supporting), writing – original draft (supporting), writing – review and editing (supporting). **Jose M. Fedriani:** conceptualization (equal), formal analysis (supporting), funding acquisition (lead), investigation (supporting), methodology (equal), project administration (lead), supervision (lead), writing – original draft (supporting), writing – review and editing (supporting).

## Conflicts of Interest

The authors declare no conflicts of interest.

## Supporting information


Data S1.

## Data Availability

The data and materials underlying this article are available in the article and in its Supporting Information.
